# Inflammation in smokers and non-smokers during implant surgery among Indians

**DOI:** 10.6026/97320630019510

**Published:** 2023-04-30

**Authors:** Raguladhithya EP, Sahana Selvaganesh, Thiyaneswaran Nessapan, Durairaj Sekar, Vishnu Priya Veeraraghavan, Rajalakshmanan Eswaramoorthy

**Affiliations:** 1 Department of Implantology, Saveetha Dental College and Hospitals, Saveetha Institute of Medical and Technical Science (SIMATS), Saveetha university, Chennai 600077, India; 2 Department of Biochemistry, Saveetha Dental College and Hospitals, Saveetha Institute of Medical and Technical Science (SIMATS), Saveetha university, Chennai 600077, India; 3 Department of Biomaterials, Centre of Molecular Medicine and Diagnostics (COMManD), Saveetha Dental College and Hospitals, Saveetha Institute of Medical and Medical and Technical Sciences, Saveetha University, Chennai 600077, India

**Keywords:** Implants, smokers and non-smokers, inflammatory biomarkers, Pro-inflammatory cytokines (IL-6, and STAT-3)

## Abstract

One of the main drawbacks faced by the dental implant surgeons is to assess the healing of the tissues and implant success for patients who are smokers. It is of interest to evaluate inflammatory biomarkers to understand the soft and hard tissue healing between smokers and non-smokers based on levels of IL-6 and STAT-3. This study included totally 20 patients (Group 1 : smokers (n=10) and Group 2: non-smokers (n=10)) undergoing stage-1 implant surgery and collected a tissue sample for the patients to assess the levels of IL-6 and STAT-3. The results indicated that there is a pronounced increase in the biomarkers in patients who are smokers in comparison to non-smokers.

## Background:

Dental implants are artificial tooth roots that are surgically placed into the jawbone to provide a foundation for replacement teeth [1]. They are commonly made of titanium or other materials that are biocompatible to the human body [2].The dental implant procedure typically involves several steps, including a consultation with a dentist or oral surgeon, an evaluation of the patient's oral health and medical history, and a customized treatment plan [2]. The dental implant itself is surgically placed into the jawbone, where it fuses with the bone tissue in a process called osseointegration [3]. This typically takes several months to complete. Once the implant has fully fused with the jawbone, a small connector post called an abutment is attached to the implant [4]. This serves as a support for the replacement tooth or teeth, which are typically custom-made to match the colour and shape of the surrounding natural teeth [5,6]. The replacement teeth are then attached to the abutment, completing the dental implant procedure. Dental implants offer several benefits compared to traditional tooth replacement options such as dentures or bridges. They are more comfortable and natural-looking, and they do not require the alteration or removal of adjacent teeth [5]. They also help to preserve the bone tissue in the jaw [7], which can deteriorate over time when teeth are missing. However, not everyone is a candidate for dental implants. Factors such as overall health, oral health, and bone density in the jaw must be evaluated to determine if dental implants are a viable option [9]. Additionally, the cost of dental implants can be higher than other tooth replacement options, and insurance coverage may be limited. Overall, dental implants can provide a long-lasting and effective solution for tooth loss, but it's important to consult with a qualified dental professional to determine if they are the right choice for your specific needs and circumstances [9, 10]. IL-6 (interleukin-6) and STAT-3 (signal transducer and activator of transcription 3) are both inflammatory biomarkers that are associated with inflammation and tissue damage. Smoking has been shown to increase levels of IL-6 and STAT-3, indicating a greater degree of inflammation in smokers compared to non-smokers [11,12]. For example, you could measure the levels of IL-6 and STAT-3 in both smokers and non-smokers before and after a surgical procedure, and then monitor their levels over time to see how they change. Other biomarkers that are involved in the inflammatory response should also be considered to get a more comprehensive picture of the healing process [14].

## Material and methods:

**Figure 1:**
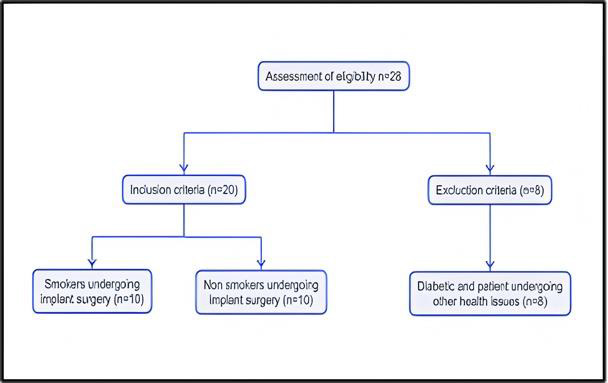
Represents the inclusion and exclusion criteria for tissue collection.

Tissue sample collection of smokers and non-smokers: Tissue samples are harvested during implant stage 1 surgery (Figure 1) using tissue punch (A minimally invasive technique) from 10 smokers and non-smokers.

## Isolation of RNA:

[1] Homogenization or lysis of the sample to release the RNA and other cellular components [14,15]. 

[2] Addition of a denaturant or chaotropic agent, which disrupts the cellular structure and facilitates RNA binding to a silica-based column or resin. 

[3] Binding of the RNA to the column or resin, and removal of other cellular components by washing with various buffers. 

[4] Elution of the RNA from the column or resin in a low-salt buffer, and quantification of the RNA using a spectrophotometer or fluorometer. 

## Quantification of RNA using nanodrop:

The Nanodrop instrument passes a beam of UV-Vis light through the RNA sample, which causes the RNA molecules to absorb the light in a characteristic manner. The amount of light absorbed by the RNA molecules is directly proportional to their concentration in the sample. The Nanodrop software uses this information to calculate the concentration of RNA in the sample, which is reported in units of ng/µl. In addition to quantifying RNA concentration, Nanodrop can also measure the purity of the RNA sample by calculating the A260/A280 ratio. This ratio is an indicator of the presence of contaminants, such as proteins or other organic compounds, in the RNA sample[16].

## cDNA synthesis:

Thermal cycler is a commonly used technique in medical research to convert RNA molecules into complementary DNA (cDNA) molecules. The process involves the reverse transcription of RNA into cDNA using a thermal cycler machine. Here are the general steps involved in cDNA synthesis by thermal cycler [17].

RNA isolation: The RNA is first isolated from the biological sample using standard RNA extraction protocols. Reverse transcription reaction: The RNA is then converted into cDNA using a reverse transcriptase enzyme and primers that are specific to the gene of interest. The reaction mix contains reverse transcriptase enzyme, random hexamer primers or gene-specific primers, and nucleotides. The mixture is incubated at an appropriate temperature and time to allow the reverse transcription to occur. PCR amplification: After cDNA synthesis, PCR amplification is performed using specific primers to amplify the gene of interest. The PCR reaction is carried out using a thermal cycler machine which allows for temperature control and specific cycling conditions that are optimized for the primers and the PCR enzyme [18].

## Gene expression analysis using QRT-PCR:

**Figure 2:**
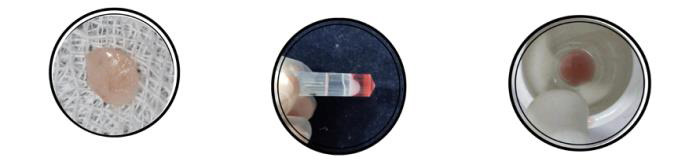
Figure represents processing of tissues and analysis of gene expression

**Figure 3:**
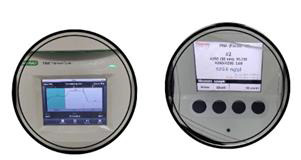
Figure represents processing of tissues and analysis of gene expression

The qRT-PCR reaction is carried out using a thermal cycler machine, which allows for temperature control and specific cycling conditions. The reaction mix contains the cDNA template, gene-specific primers, fluorescent probes, and a qPCR enzyme. The reaction mixture is amplified by cycling through different temperatures, and the fluorescent signal is monitored in real-time. Data analysis: The data obtained from qRT-PCR is analyzed using various software programs that allow for the quantification of the amount of RNA transcript present in the sample (Figure 2,Figure 3). The results are usually presented as Quantitative real-time PCR (qRT-PCR) is a commonly used technique in medical research for gene expression analysis. It is a sensitive and accurate method for quantifying RNA transcripts in a biological sample and it can be used to measure changes in gene expression levels under different experimental conditions. Here are the general steps involved in qRT-PCR gene expression analysis: RNA isolation: The RNA is first isolated from the biological sample using standard RNA extraction protocols. cDNA synthesis: The RNA is then reverse transcribed into cDNA using a reverse transcriptase enzyme and primers that are specific to the gene of interest. The cDNA synthesis can be done by either one-step or two-step reactions. Fold-change in gene expression levels between the different experimental conditions is known [19].

## Results and Discussion:

**Figure 4:**
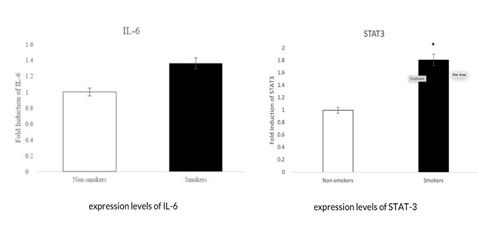
represents the result of IL-6 and STAT 3

The results of samples collected show high risk in smokers compared to non-smokers. Smoking has been known to have detrimental effects on oral health, including the success rate of dental implants. Recent studies have shown that smokers have higher levels of inflammatory biomarkers, such as STAT-3 and IL-6, compared to non-smokers [20]. These inflammatory markers can aggravate swelling and secondary infection after the placement of dental implants, which can result in implant failure. The expression of STAT-3 and IL-6 is known to be higher in smokers due to the harmful chemicals found in cigarette smoke (Figure 4). These chemicals can damage the immune system, impair the body's ability to fight off infections and lead to chronic inflammation. This chronic inflammation can interfere with the normal healing process of the implant, resulting in slower and less predictable Osseo integration, as well as an increased risk of implant failure. Therefore, it is recommended that smokers reduce or quit smoking altogether to improve their chances of successful implant placement and long-term implant survival. By reducing the levels of inflammatory biomarkers in the body, smokers can help promote a healthier and more favorable environment for implant healing. Additionally, smoking cessation can also improve overall oral health, reduce the risk of oral cancer, and improve general health outcomes.

## Conclusion:

Peri-implantitis is a serious condition that can lead to implant failure and significant patient morbidity [20]. Recent studies have shown that patients with peri-implantitis have increased expression of pro-inflammatory cytokines, such as IL-6 and STAT-3. This suggests that chronic inflammation plays a significant role in the development and progression of peri-implantitis. Smokers are at an increased risk of developing peri-implantitis due to the harmful effects of smoking on the immune system and the body's ability to heal [14]. As such, it is important to provide special care to smoker patients after the placement of the implant. This includes prescribing mouthwash and educating patients on the importance of oral hygiene and the potential complications of smoking. Thus, pro-inflammatory cytokines such as IL-6 and STAT-3 are often expressed in the tissue of patients with peri-implantitis, highlighting the importance of preventing chronic inflammation in implant patients. Special care should be taken with smokers, who are at an increased risk of developing peri-implantitis. Providing education and prescribing mouthwash can help reduce the risk of complications and promote successful implant healing.
